# VISDB: a manually curated database of viral integration sites in the human genome

**DOI:** 10.1093/nar/gkz867

**Published:** 2019-10-10

**Authors:** Deyou Tang, Bingrui Li, Tianyi Xu, Ruifeng Hu, Daqiang Tan, Xiaofeng Song, Peilin Jia, Zhongming Zhao

**Affiliations:** 1 Center for Precision Health, School of Biomedical Informatics, The University of Texas Health Science Center at Houston, Houston, TX 77030, USA; 2 School of Software Engineering, South China University of Technology, Guangzhou, Guangdong 510006, P.R. China; 3 Department of Biomedical Engineering, Nanjing University of Aeronautics and Astronautics, Nanjing, Jiangsu 211106, P.R. China; 4 Human Genetics Center, School of Public Health, The University of Texas Health Science Center at Houston, Houston, TX 77030, USA; 5 MD Anderson Cancer Center UTHealth Graduate School of Biomedical Sciences, Houston, TX 77030, USA; 6 Department of Biomedical Informatics, Vanderbilt University Medical Center, Nashville, TN 37203, USA

## Abstract

Virus integration into the human genome occurs frequently and represents a key driving event in human disease. Many studies have reported viral integration sites (VISs) proximal to structural or functional regions of the human genome. Here, we systematically collected and manually curated all VISs reported in the literature and publicly available data resources to construct the Viral Integration Site DataBase (VISDB, https://bioinfo.uth.edu/VISDB). Genomic information including target genes, nearby genes, nearest transcription start site, chromosome fragile sites, CpG islands, viral sequences and target sequences were integrated to annotate VISs. We further curated VIS-involved oncogenes and tumor suppressor genes, virus–host interactions involved in non-coding RNA (ncRNA), target gene and microRNA expression in five cancers, among others. Moreover, we developed tools to visualize single integration events, VIS clusters, DNA elements proximal to VISs and virus–host interactions involved in ncRNA. The current version of VISDB contains a total of 77 632 integration sites of five DNA viruses and four RNA retroviruses. VISDB is currently the only active comprehensive VIS database, which provides broad usability for the study of disease, virus related pathophysiology, virus biology, host–pathogen interactions, sequence motif discovery and pattern recognition, molecular evolution and adaption, among others.

## INTRODUCTION

Viral infection causes a variety of human diseases including cancer (1–6). Viruses invade host cells and interact with host molecules, thereby potentially interrupting normal function of the host cells and causing disease. Previous studies have linked hepatitis B virus (HBV) with hepatocellular carcinoma (HCC) (1,7); human herpesvirus (HPV) with cervical carcinoma, squamous-cell carcinoma and head and neck squamous cell carcinoma (HNSC) (2); Merkel cell polyomavirus (MCV) with Merkel cell carcinoma (3); Epstein–Barr virus (EBV) with nasopharyngeal carcinoma (4); human T lymphotropic virus type 1 (HTLV-1) with adult T-cell leukemia (5) and adeno-associated virus type 2 (AAV2) with HCC (6). Since oncogenic viruses associate with 15% of tumor cases overall (8), a comprehensive resource for viral integration sites (VISs) is crucial to significantly increase our understanding of the molecular mechanisms underlying tumorigenesis. Such a resource will facilitate systematic analysis of VISs providing valuable insight into the mechanisms of viral integration into host genomes or gene sequences. These systematic analyses will potentially lead to prevention of viral infection, enhanced screening for susceptible populations, and development of novel therapeutic strategies for personalized treatment.

Viruses directly transform infected cells by affecting gene mutation/genomic alteration, gene expression, pathway interaction and chromosome instability (9). For example, HBV integration into the *SERCA1* gene disturbs endoplasmic reticulum calcium homeostasis and induces apoptosis (10). Inactivation of p53 or depletion of its function in infected cells causes genomic instability and interference with DNA repair mechanisms (11). The integration of the tumor suppressor gene *RB1* with the HPV E7 gene results in driving infected quiescent cells back into a proliferative state to cause viral genome replication. Moreover, viruses can also transform cell activity via chronic infection and inflammation. A well-known example is HCC induced by chronic HBV infection, where HBV integration plays a critical role in tumorigenesis (12).

Non-coding RNA (ncRNA) plays a critical role in viral infection. Increasing evidence has shown that microRNAs (miRNAs) are involved in RNA silencing and post-transcriptional regulation of gene expression (13). Viral insertion events can potentially affect the miRNA binding region in the host genome. Moreover, viral miRNAs can regulate both cellular and viral gene expression to establish a host environment conducive to the completion of the viral life cycle. For example, Lars *et al.* used luciferase assays with sensor vectors carrying the 3′ untranslated regions (UTRs) of TOMM22 and IPO7 to demonstrate significant repression by ebv-miR-BART16 and ebv-miR-BART3, respectively (14).

Many experimental approaches have been developed to identify VISs including fluorescence *insitu* hybridization, amplification of papillomavirus oncogene transcript assay and various extensions of polymerase chain reaction (PCR). Next generation sequencing (NGS) combined with PCR is currently a typical approach to detect and validate VISs or other types of mutations (15). Recently, several methods have been developed for VIS detection from NGS data such as HIVID (16), VirusFinder (17), VERSE (18) and ViFi (19), accelerating the process of VIS discovery.

Many VISs have been detected during the past two decades. However, these VISs are unsystematically distributed across the literature or public databases. Critically, those VISs were derived from different viruses and different versions of the human reference genome, making it difficult for investigators to systematically search, compare and analyze VIS data. Since 2013, four databases, Dr.VIS (20), RID (21), HPVbase (22) and HIRIS (23), have been developed to address the aforementioned problems. However, these resources have the following limitations. HPVbase and HIRIS are restricted to information on only a single virus, HPV and HIV, respectively. RID is limited to information on three human retroviruses, and the majority of data were based on computational prediction. Dr.VIS is a disease-related VIS database covering eight viruses, but its website has been unavailable, and the information content is also limited (e.g. only 1453 VISs). Furthermore, most VIS databases have not been updated for several years, while NGS technology has dramatically advanced the discovery of VISs in recent years. Thus, a comprehensive, carefully curated and updated VIS database is needed to advance biomedical research.

In this study, we present a manually curated database, called the **V**iral **I**ntegration **S**ite **D**ata**B**ase (VISDB, available at https://bioinfo.uth.edu/VISDB). VISDB represents the most comprehensive collection of VISs from DNA viruses (26 897 integration sites) and RNA retroviruses (50 735 integrations sites). All these VISs were extracted from the original studies and manually curated. The current version of VISDB does not contain any computationally predicted VISs. Literature derived, genome-annotation and target gene information data were successively curated and integrated into VISDB. Web tools were developed and implemented to visualize VISs, VIS clusters, VIS proximate regions and VIS-involved ncRNA-associated interactions, among others. A user-friendly web interface was developed to allow users to browse, search, curate, analyze, visualize and download data. Furthermore, VISs can be browsed by features such as completeness (a method we developed for evaluating the quality of each VIS), virus, disease, method, chromosome fragile site (CFS), literature, target gene, miRNA and genomic region.

## DATABASE AND ANALYSIS

### Overview

**Figure 1.**
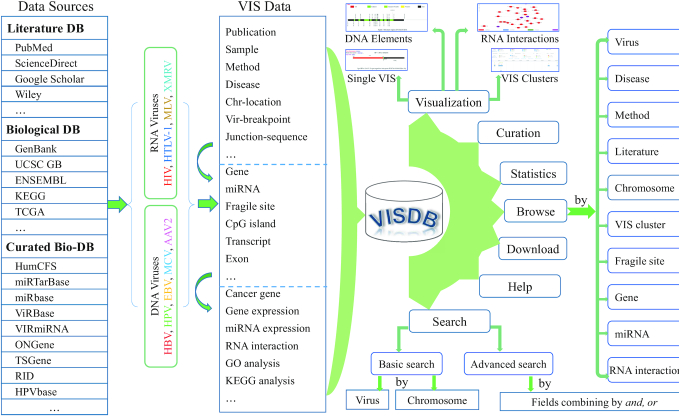
Overview of VISDB. Data were collected from three sources (literature, biological DB and curated Bio-DB) and curated into three categories. The core part is the identification and curation of the VISs. Various functions are implemented in VISDB.

Figure 1 illustrates the overview of VISDB. Data were obtained from three different sources: (i) literature, (ii) biological databases and (iii) curated biological databases. First, VISs were manually extracted from the literature and subsequently annotated with additional information including viral sequences, metabolic pathways, target genes, nearby genes, miRNAs, CFSs, transcript start sites (TSSs), CpG islands, integrated virus sequences and target human sequences. Next, target genes were annotated using data from other biological databases, including oncogene and tumor suppressor gene status, viral and cellular ncRNA-associated virus-host interactions and gene ontology (GO) annotations. Based on target genes and cancer data downloaded from The Cancer Genome Atlas (TCGA), the expression of VIS target genes and potentially regulated miRNAs was analyzed. In particular, differential expression between cancer and normal tissue in five cancer types [cervical squamous cell carcinoma and endocervical adenocarcinoma (CESC), HNSC, liver hepatocellular carcinoma (LIHC), prostate adenocarcinoma (PRAD), and stomach adenocarcinoma (STAD)] was evaluated. VISDB provides intuitive visualization of virus integration events, DNA elements around VIS, VIS clusters and virus-host ncRNA-associated interactions, which involve the target genes. Our user-friendly web tool has functions such as browse, search, curation, gene feature, miRNA feature, statistical analysis and download.

We refer to genes harboring at least one VIS as target genes. VIS can fall into any gene region including 5′UTR, exon, intron and 3′UTR. If one VIS is located in more than one gene (some genes overlap), we consider all of these genes as target genes. We define a ‘VIS cluster’ as a set of VISs belonging to the same functional or structural region. VISDB offers various levels of granularity to define these regions including cytoband, gene/intergenic region, transcript/intergenic region and exon/intergenic region. Intergenic regions are characterized using genomic coordinates.

### Data source and data collection

The data underlying VISDB was obtained from an extensive literature search using keywords and mining publicly available biological databases. Scientific literature describing VISs was downloaded from PubMed, ScienceDirect, Google Scholar and Wiley. Additionally, some VISs were downloaded from RID and HPVbase.

We integrated gene annotations from NCBI GenBank and ENSEMBL to curate target and nearby genes. GTF files and enhancer/promoter annotations were downloaded from the UCSC Genome Browser and GeneHancer (24) to define DNA elements such as transcript, exon, enhancer and promoter regions. Information on viral metabolic pathways and genes in metabolic networks were obtained from the Kyoto Encyclopedia of Genes and Genomes (KEGG). CFS and CpG island data were downloaded from HumCFS (25) and UCSC Genome Browser, respectively. We also integrated miRNA data from miRTarBase (26) and miRbase (27) and collected virus-host ncRNA-associated interactions from VIRmiRNA (28) and ViRBase (29). Oncogene and tumor suppressor gene annotations were derived from ONGene (30), TSGene (31,32) and KEGG. Cancer expression data were downloaded from TCGA. Virus and target human sequences were extracted from the NCBI Nucleotide database.

We searched for VIS literature using the following keywords: virus integration, viral integration, integration, integration site, full name of virus, abbreviation of virus, etc. After manually curating the literature and corresponding supplementary data, we removed articles without exact integration sites.

VIS data were extracted and curated from the original publications manually. SQL, Perl and PHP programs or scripts were implemented to refine high quality VIS data. In addition, string operation (concatenate, find, left, right and length), sort, search and replace functions were used to extract information, remove duplicates or group the rows to accelerate data compilation. To avoid inconsistencies between different versions of the human reference genome, we re-mapped all VISs using the human reference assembly version GRCh38.p12.

### Data content and categories

VISDB is characterized by a comprehensive curation of samples, diseases, publications, sequences, genes, CFSs, transcripts, CpG islands, miRNAs, virus-host ncRNA-associated interactions as well as TCGA miRNA and gene expression (Table 1). As implied in Figure 1, VISDB consists of the following categories of information:

**Table 1.** Summary of VISDB and comparison with Dr.VIS 2.0 and HPVbase^a^

**Improved data items**	**HPVbase**	**Dr.VIS 2.0**	**VISDB**
# total VISs (# viruses)	1257 (1)	3340 (8)	77 632 (9)
# diseases	8	25	27
# samples	NA	3339	2880
# experimental methods	42	NA	50
# publications	59	64	108
# junction sequences	NA	551	2577
# target genes/# VISs	481/713	1153	15 064/49 525
# nearby genes/# VISs	NA	406	11 643/28 132
# nearest CFSs/# VISs	98/395	NA	123/25 193
**New annotation features**			
# virus sequences			33 066
# target sequences			76 290
# transcripts mapped with VIS’s flanking sequences			16 353
# CpG islands mapped with VIS’s flanking sequences			14 595
# miRNAs mapped with VIS’s flanking sequences			207
# genes with expression/# VISs			3594/8610
# miRNAs with expression^b^/# VISs			227/3092
**Classification of target gene**			
# oncogenes/# VISs			451/2768
# tumor suppressor genes/# VISs			680/3707
# pathway genes/# VISs			105/624
**Classification of nearby genes**			
# oncogenes/# VISs			314/940
# tumor suppressor genes/# VISs			450/1362
**Virus-host ncRNA-associated interactions**			
# VISs correlated with lncRNA-associated interaction			83
# VISs correlated with miRNA-associated interaction			26 414

^a^Two virus integration datasets/databases, RID and HIRIS, are not included in comparison because there are no such statistics from their publications or websites.

^b^We only include the miRNAs whose target genes mapped with VIS and having expression data in TCGA.

#### Level I—literature derived data

This category contains publication and sample metadata, experimental assay and disease information, viral and human genome assembly versions, VIS locations, breakpoints, flanking sequence information, etc.

We constructed a universal virus integration event model to coordinate VISs across various original publications or datasets (Supplementary Figure S1). As of June 2019, 77 632 VISs involved in five DNA oncoviruses (HBV, HPV, EBV, MCV, AAV2) and four RNA retroviruses (HIV, MLV, HTLV-1, XMRV) were curated using the aforementioned model. VISs were annotated with the detection method in the original publications (Supplementary Table S1).

As VIS information may be incomplete, we defined a ‘quality evaluation’ index to assess annotation quality using the following criteria: (i) perfect: junction sequence, viral and human reference genomes are provided to display the viral sequence integrated into the specific location on the human genome; (ii) good: precise start and end location of the integration site on chromosome, viral and human reference genomes are provided to show the patterns of the virus integration, but the sequence resulting from the integration event is missing; (iii) normal: only one precise chromosome location, viral and human reference genomes are provided to infer the location of the integration site; (iv) weak: VIS with approximate location and without the precise location site on the chromosome.

#### Level II—genome-annotation data

This category comprises genomic annotation data for the corresponding location or breakpoint, including fusion and nearby genes, CFS, nearest CpG island, nearest TSS, nearest miRNA, viral sequence and target sequence information.

Using the precise positions extracted from the literature, both upstream and downstream regions of VISs were analyzed to define the elements. Since different gene symbols or aliases may have been used in the literature, we annotated all target or nearby genes with the official gene symbol to eliminate potential inconsistencies. For example, we use *KMT2B* to replace *CXXC10, DYT28*, *MLL1B*, *MLL2*, *MLL4*, *RX2*, *TRX2*, *WBP7* and *WBP-7*. In addition, we extracted flanking sequences of the breakpoints or integration sites from NCBI when the accession number of the viral genome and precise coordinates of the VIS in the human genome were available.

#### Level III—target gene information

This category contains data annotating the VIS target genes. This information includes oncogene and tumor suppressor gene status, interaction partners, pathway and GO annotations, as well as TCGA expression patterns for the respective VIS target gene.

Gene expression and function play a significant role in genomic research. For HBV, HPV, HIV and HTLV-1, target genes with more than five VISs were used for the functional enrichment analysis. GO enrichment analysis (including Biological Processes, Cellular Component and Molecular Function domains) was performed using the online tool WebGestalt (33) (version 2019) with default parameters (false discovery rate < 0.05). We performed expression analysis of target genes and regulated miRNAs in five cancer types (CESC, HNSC, LIHC, PRAD, STAD) from TCGA. The statistical significance of the differences between the normal and cancer samples was examined by the t-test. Only miRNAs with *P*-values < 0.05 were retained.

Virus–host interactions are processes occurring during viral infection, which enable both partners to respond to each other. According to Li *et al.* (29), virus–host interactions can be divided into four categories based on the role of the virus and the host during viral infection: virus2virus, virus2host, host2virus and host2host interactions. We highlighted the subset of interaction networks with target genes involved in the interaction. The host2host, host2virus and virus2host models were used and implemented on our website to identify virus–host ncRNA-associated interactions, which always involve the target genes. The pathways of five viruses (HBV, HPV, EBV, HTLV-1 and HIV) and 368 pathway genes were curated from the KEGG database. There were 105 genes involved in 624 integration events (Supplementary Table S8).

### Web interface

**Figure 2.**
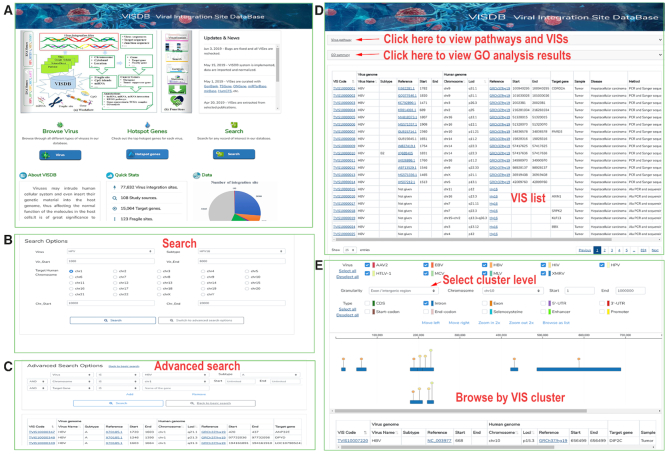
Main features of the VISDB web content. (**A**) Homepage. (**B**) Basic search. (**C**) Advanced search. (**D**) Browse function of VISs on the Virus web page. The page shows the HBV information, including reference, positions in the genome, disease and method. On the top, users can view pathway and GO analysis results. (**E**) Browse VIS data by cluster using *‘Clustered VIS browse’* submenu in Browse menu.

**Figure 3.**
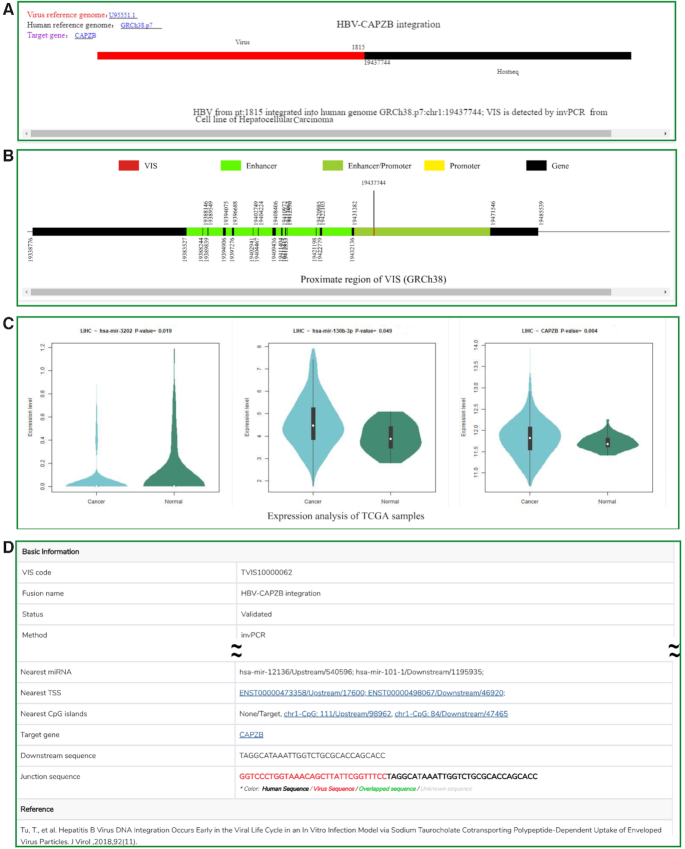
The web page showing detailed VIS analysis results. (**A**) Visualization of a single integration event. (**B**) Visualization of DNA elements flanking a VIS. (**C**) Gene and miRNA expression analysis using The Cancer Genome Atlas (TCGA) data. (**D**) Detailed description of VIS. Only selected parts of the page are shown due to space limitations.

VISDB provides several functions: browse, VIS cluster browse, search, advanced search, curation, download, statistics and help (Figures 2 and 3). All VISs can be browsed by virus name, disease name, CFS, experimental assay, chromosome, literature, gene, miRNA and virus-host interaction, respectively. All VISs can be browsed by data quality. VISs matching the user's search condition are listed in a table containing VIS-related virus genome, human genome, target gene, sample type, sample-related disease, method and source literature (Figure 2D). The detail page is composed of four panels: single VIS integration event (Figure 3A), proximate DNA element visualization (Figure 3B), TCGA gene and miRNA expression visualization (Figure 3C) and description (Figure 3D).

The basic search function allows the user identify locating VIS by virus or chromosome coordinate range (Figure 2B), while the advanced search provides the user an option of finding VIS related information based on custom parameters (Figure 2C). The virus page is comprised of virus metabolic pathways and networks, target gene GO analysis and list of VISs of the specific virus (Figure 2D). Furthermore, the curation page allows contributors to submit or supplement VIS data to VISDB. The download page enables access to all curated data in VISDB. The statistics page shows a summary of VISs on chromosomal distribution, target genes and CFSs. The help page describes the VISDB framework and provides user guidance, etc.

### Visualization

VISs, VIS clusters, DNA elements around VISs and virus–host ncRNA-associated interactions related to VISs can be visualized using the GRC38h.p12 reference genome using a computer program we developed. VISs reported using older versions of the reference genome were re-mapped to GRC38h.p12.

#### Visualization of single integration event

Integration of viral sequence into the human genome leads to the generation of junction sequences containing nucleotides from both the virus and the target human sequence. We specify each VIS event by combining the virus name with the genome information including gene symbol, cytoband or chromosome, depending on the completeness of the VIS. Different colors specify viral sequences, human sequences, overlapping sequences and unknown sequences. Coordinates of a single VIS in the viral genome and host genome are provided. Target gene and human reference genome are listed, and links are added to all elements in the plot. Description of the integration event is created by combining the virus, breakpoints, integration sites and target gene (Figure 3A). So far, 1651 VISs with junction sequences can be visualized perfectly after we manually labelled the junctions with local coordinates.

#### Visualization of DNA elements around VIS

For each VIS with only a single insertion site, we visualize the region 25 kbp upstream and downstream. For VISs with insertion start and end locations, we visualize the region 25 kbp upstream of the start location and 25 kbp downstream of the end location. Colors distinguish different DNA elements. The coordinates are marked under the boundary of each element (Figure 3B). Our analysis indicated that 92.0% of VISs (70 345/76 447) harbored DNA elements in the proximity, with an average of 7.7 functional elements per region.

#### Visualization of VISs in a specific region

We annotate VIS clusters with four levels of information: cytoband, gene and intergenic region, transcript and intergenic region, exon/intron/enhancer/promoter/CDS/start codon/stop codon/5′-UTR/3′-UTR and intergenic region. All VIS clusters can be browsed in the ‘list’ or ‘plot’ mode. In the list mode, all valid VIS clusters are listed in a table and the frequency of each VIS cluster is calculated by dividing the number of VISs in the current cluster by the total number of VISs involved in the selected region. In the plot mode, VISs belonging to the same cluster are presented as differently colored nodes, which present different virus types. Detailed information is displayed upon hovering over the node. A list of VISs is shown at the bottom upon clicking on the colored node (Figure 2E).

#### Visualization of interaction networks related to VIS

The virus–host ncRNA-associated interaction networks of EBV, HBV, HIV, HPV, HTLV-1 and MCV are visualized with Cytoscape (34). We only retained virus2host, host2host and host2virus interactions to focus on the relationship between viral infection and VISs. All VISs involved in the interactions are listed at the bottom and hyperlinks to the original source are provided (Supplementary Figure S2).

### Data summary

#### Chromosomal distribution of VIS

Previous studies have reported that VISs are randomly distributed across all chromosomes (35). Our data support this conclusion when using VISs of all viruses or each individual virus (Supplementary Figure S3A). However, after normalizing VIS numbers on each chromosome by chromosome length or the total length of all chromosomes, we found that VIS densities of chr19, chr17 and chr16 were 1.85-, 1.59- and 1.31-fold higher than the genome-wide average, respectively (Supplementary Figure S3B). Specifically, HBV had a hotspot on chr19 (top three hotspot genes are *KMT2B*, *CCNE1* and *PRPF31*) and chr5 (hotspot genes *TERT* and *LOC110806263*); HIV had a hotspot on chr19 (hotspot genes *HNRNPM* and *CEACAM21*) and chr17 (hotspot genes *RPTOR* and *IKZF3*).

When we restricted the region to cytoband and gene/intergenic locus, our dataset showed that 29.9, 28.3, 24.6, 29.8 and 29.9% of cytobands harbored VISs for HBV, HPV, EBV and HTLV-1, respectively. Moreover, 50% of VISs located in 20% of VIS-targeted cytobands (Supplementary Table S2), indicating that VISs are not randomly distributed in the genomes. Here, we listed a few hotspot regions: 19q12-q13.12, 5p15.33 and 2p11.2 form the hotspot cytoband of HBV integration; 21q22.2–22.3, 18q22.1, 8q24.21 and 11q14.3 form the hotspot cytoband of HPV integration; 9p13.3, 20q13.2, 19p13.2 and 16q22.1 form the hotspot cytoband of HIV integration; and 5q21.1–22.1, 14q12, 17q11.2 and 2p12 form the hotspot cytoband of HTLV-1 integration.

CFSs are regions which are subject to genome change and hotspots for DNA rearrangement, deletion and recombination at an increased likelihood (36). Our dataset showed that 33.9% of HBV, 37.5% of HPV, 42.2% of HIV, 34.6% of HTLV-1 and 35.2% of EBV integration sites were observed in chromosome fragile regions, respectively (Supplementary Table S3). The heatmap of the distribution of CFSs showed that most CFSs were targeted by five viruses, whose hotspots are FRA2S, FRA1A. FRA19A and FRA19B. However, FRA10E and FRA16C were only specifically targeted by HBV and HTLV-1, respectively (Supplementary Figures S4 and 5).

#### Target genes

Recent studies reported that 45–47% of HBV integrations (35) are gene-targeted. Our collected data shows that ∼55% of VISs of HTLV-1, HBV, HPV and EBV fall into the gene body compared to 82.3% of HIV VISs (Supplementary Table S4), suggesting that HIV tends to target genic region at a higher frequency than other viruses. Interestingly, when we extended functional regions to include enhancers and promoters, we found VISs located in gene/enhancer/promoter regions accounted for 66.0% of total VISs (Supplementary Table S5). This result suggested that viruses preferentially integrate into functional regions of the genome.

Oncogenes and tumor suppressor genes are found to have a higher probability to be targeted than other genes (Supplementary Table S6). We also calculated the average VIS number located in oncogenes, tumor suppressor genes and other genes. Statistical analysis show that there are on average 6.13, 5.43 and 3.12 VISs located in these three types of genes, respectively. These results suggest that oncogenes and tumor suppressor genes are preferentially targeted (Supplementary Table S7). Statistical analysis of hotspot genes indicates that *TERT*, *FN1* and *KMT2B* have high chance of integration by HBV. *FHIT* is integrated more frequently than other genes by HPV. *MRTFB*, *BACH2* and *HORMAD2* are targeted preferentially by HIV. HTLV-1 integration has no obvious preference on genes (Supplementary Figure S6).

Furthermore, we analyzed genes reported in virus metabolic pathways and its association with VISs. Genes reported to be involved in virus pathways harbored DNA VISs at a lower frequency than expected (10.8%). Strikingly, among the 114 EBV pathway genes, there was only one VIS, which occurred on *PLCG2* (0.9%). RNA retroviruses, on the other hand, showed enrichment of VISs in genes reported to be involved in virus pathways (37.5%). This simple analysis suggested that DNA virus integration might not affect metabolism directly (Supplementary Table S8).

#### VIS and other functional regions

Previous studies have shown that virus integration occurs preferentially near TSSs (37). Therefore, we calculated the distance between a VIS and its nearest TSS. We observed that HIV integration occurred more frequently in a 10 kb region within the TSS compared to HBV, HPV and HTLV-1. Furthermore, about 70% of four viruses' integration sites were located within 50 kb flanking the TSS (including TSS). In contrast, HIV integration in this region was higher than 90%.

We further identified the closest CpG islands and miRNAs on the flanking region of the four viruses’ integration sites. Our dataset showed that <20% of VIS located within the upstream and downstream 10 kb region of CpG islands. Even when we extended the distance to 50 kb, the percentages were <50% except HIV. Our dataset also showed that <2.5% of VIS located in 10 kb region around miRNAs, which was consistent with our expectation (Supplementary Figure S7).

## CONCLUSION AND FUTURE DEVELOPMENT

Studies have shown that virus integration can cause genomic instability and serious damage to the host genome. However, knowledge such as the correlation of integration patterns with phenotype and the spatial and temporal effects of integration events is still unclear. In this work, we systematically curated the virus integration events on the human genome. We collected the data from public data sources and publications to provide the insights for research communities on VISs, and to have a better understanding of how virus affects the phenotype of the host. To serve the broad biomedical research community, we will continuously update our VISDB and routinely expand the database with more viruses and features, especially NGS is now a routine research approach. We will expand VISDB to include VISs in other animal models. Currently, in our statistical analysis, we are mainly focused on the integration events on the oncogenes. We will examine more diseases or phenotypes that are affected by virus. We will develop more computational tools based on our collected data to calculate the integration patterns, predict the VISs and evaluate the data quality, and provide such tools free for the research communities.

## Supplementary Material

gkz867_Supplemental_File
