# Supplementary material for: VISDB: a manually curated database of viral integration sites in the human genome
Source: Nucleic Acids Res. 2019 Oct 10;48(D1):D633–41. doi: 10.1093/nar/gkz867 (PMC6943068; doi:10.1093/nar/gkz867)
Supplement: gkz867_Supplemental_File [file gkz867_supplemental_file.pdf]

VISDB Supplementary file

Table S1. Statistics of curated VISs in VISDB

Table S2. The ratios of cytobands harboring VISs

Table S3. The ratios of VISs located at chromosome fragile sites

Table S4. The ratios of VISs located in genes

Table S5. VIS distribution on DNA elements

Table S6. Ratios of oncogenes, tumor suppressor genes and non-classified genes targeted by VIS

Table S7. Average VIS number located in genes

Table S8. Ratios of pathway genes targeted by VIS

Figure S1. Virus integration event model

Figure S2. Visualization of virus-host interactions involved in miRNAs

Figure S3. Chromosomal distribution of VISs

Figure S4. Heatmap of CFS after normalization of VIS number

Figure S5. Heatmap of CFS percentage for HBV, HPV, HIV, HITLV-1 and EBV

Figure S6. Top 25 most frequently targeted genes of four viruses: HBV, HPV, HIV and HTLV-1

Figure S7. Cellular function regions in the vicinity of VISs

Tables

Table S1 shows the statistics of VISs in VISDB. 77 632 VISs involved in 5 DNA oncoviruses (HBV, HPV, EBV, MCV, AAV2) and 4 RNA retroviruses (HIV, MLV, HTLV, XMRV) are curated from 108 publications. All VISs are evaluated by the completeness of VIS data and further categorized according to the method used to detect VIS in the original articles.

Table S2 shows the statistics of cytobands harboring VISs.

Table S3 shows the results of chromosome fragile sites harboring VISs in HBV, HPV, HIV, HTLV-1 and EBV integration.

Table S4 shows the frequency of VISs located in gene and intergenic regions.

Table S5 shows the frequency of VISs located in exons, intron, promoter regions, etc.

Table S6 shows the target rate of three kinds of genes.

Table S7 shows the average VIS number located in genes. The top 25 most frequently targeted genes of HBV, HPV, HIV and HTLV-1 integration are shown in Figure S6.

Table S8 shows the statistics of pathway genes targeted by VISs.

Table S1. Statistics of curated VISs in VISDB

Virus	# VIS	# C1£	# C2£	# C3£	# C4£	# methods	# diseases	# samples	# papers*
HBV	20 588	936/296	102/1983	768/15 990	397/116	22	2	1711	45
HPV	5118	385/9	125/9	311/3900	325/54	19	23	676	31
EBV	1144	12/2	2/0	0/1098	0/30	7	6	40	7
MCV	55	0/0	3/1	11/6	34/0	6	3	51	9
AAV2	24	11/0	0/3	0/10	0/0	4	1	21	3
HIV	16 797	0/0	2887/0	772/13 138	0/0	6	4	58	10
MLV	32	0/0	0/0	32/0	0/0	1	-	32	1
HTLV-1	33 845	0/0	0/0	0/33845	0/0	1	2	272	4
XMRV	29	0/0	0/0	29/0	0/0	2	1	19	2
Total	77 632	1344/307	3119/1996	1923/67 987	756/200	50	27	2880	108

£ C1—VISs curated with all genomics annotations, junction sequence and site-adjacent sequence; C2—VISs with two precise integrated locations are curated with all genomics annotations and target sequence; C3—VISs with one precise integrated location are curated with all genomics annotations and site-adjacent sequences; C4—VISs only with rough integrated location. Numbers before and after “/” are VISs detected by wet lab assay and non-wet lab assay.

* Some articles harboring multiple kinds of viral integration.

Table S2. The ratios of cytobands harboring VISs

	HBV	HPV	EBV	HIV	HTLV-1
#Cytoband	258	244	212	257	258
Percentage	29.9%	28.3%	24.6%	29.8%	29.9%

Note: 862 cytobands are retained to cluster and calculate VIS distribution on cytobands after discarding cytobands that do not belong to chr1-chr22, chrX and chrY with dataset downloaded from UCSC Genome Browser.

Table S3. The ratios of VISs located at chromosome fragile sites

	HBV	HPV	HIV	HTLV-1	EBV
# VISs located in CFS	6761	1749	7088	11 711	388
Total VISs with precise location	19 953	4666	16 790	33 842	1102
Percentage	33.9%	37.5%	42.2%	34.6%	35.2%

Table S4. The ratios of VISs located in genes

Virus	# VISs in introns	# VISs in intergenic regions	Ratio
EBV	598	504	54.3%
HBV	10 270	9683	51.5%
HIV	13 812	2979	82.3%
HPV	2721	1967	58.0%
HTLV-1	19 016	14 826	56.2%

We only calculated VIS with precise chromosome location.

Table S5. VIS distribution on DNA elements

Region type	# VISs	Target rate
CDS	1858	2.4%
Enhancer	11 496	15.0%
Exon	4871	6.3%
5' UTR	436	0.6%
Intergenic	29 999	39.1%
Intron	44 091	57.5%
Promoter	39	0.1%
Promoter/Enhancer	3857	5.0%
Start_codon	75	0.1%
Stop_codon	96	0.1%
3' UTR	1856	2.4%
Gene+Enhancer+Promoter	50 673	66.0%

76 724 VISs with valid chromosome location are used to calculate the ratios.

Table S6. Ratios of oncogenes (OG), tumor suppressor genes (TSG) and non-classified genes targeted by VISs

	OG	TSG	ALL
#Reference gene	803	1217	61 316
#Target Gene	451	680	15 064
Target rate	56.2%	55.9%	25.4%

Table S7. Average VIS number located in genes

	OG	TSG	ALL	Non-tumor
#VIS	2765	3698	49 525	43 459
#Target Gene	451	680	15 064	13 944
Average VIS number	6.13	5.44	3.29	3.12

OG: oncogene; TSG: tumor suppressor gene.

Table S8. Ratios of pathway genes targeted by VIS

	HBV	HPV	HIV	HTLV-1	EBV	DNA virus	RNA virus
Total Pathway genes	102	118	128	120	114	334	248
Total Pathway genes targeted by VIS	23	12	52	41	1	36	93
Ratio	22.5%	10.2%	40.6%	34.2%	0.9%	10.8%	37.5%

Viral integration site data model

A universal virus integration event model is constructed to coordinate viral integration sites across various original publications or datasets. The model covers detection, analysis and archival of viral integration sites (VIS), allowing junction sequences to show complicated cases of viral integration such as rearrangement, mutation, reverse inserts and microhomology junctions. The model consists of four elements: basic information, virus sequence, target host sequence and junction sequence (Figure S1). The category of basic information includes sample type and detection or description methods for VIS in the original article. In addition, some identifying attributes such as a status attribute are added to show whether the VIS is detected by an experimental assay and a completeness attribute to evaluate the integrity of VIS.

The junction sequence category has the most significant role in downstream analysis. Virus integrates to human genome may have many different patterns. The simplest pattern is a segment of the virus sequence is broken and inserted into the host's genome without any other process in the occurrence of integration event. However, reverse-inserts, rearrangements, microhomology and mutations may take place in the process of integration, and the integration event may be complex. Therefore, we consider a virus-integrated within a human sequence to have the form of "human sequence" + "virus-mixed sequences" + "human sequence". In other words, a junction sequence is composed of a human sequence preceding the integrating region, a sequence mixed with virus sequences and unknown sequences excluding human sequences, and a human sequence following the integration region. Notably, overlap of human sequence, virus sequence and unknown sequence between human sequence and virus sequence are both allowed. However, no human sequence can exist in the mixed sequence; otherwise, the integration event is divided into two events.

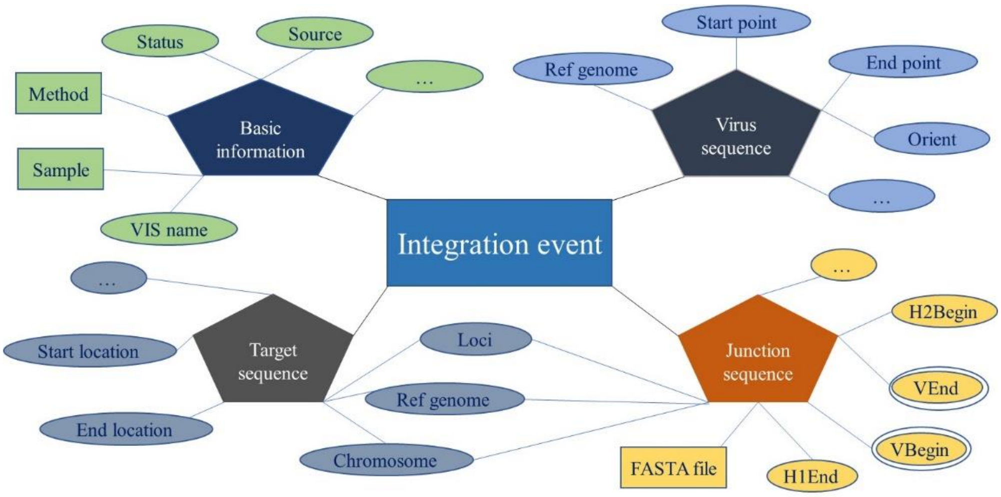

Figure S1. Virus integration event model.

Visualization of virus-interaction related to VIS

We use API provided by Cytoscape to visualize the interactions (Figure S2). The number of VISs involved in lncRNA-associated interaction and miRNA-associated interaction are 83 and 26 414.

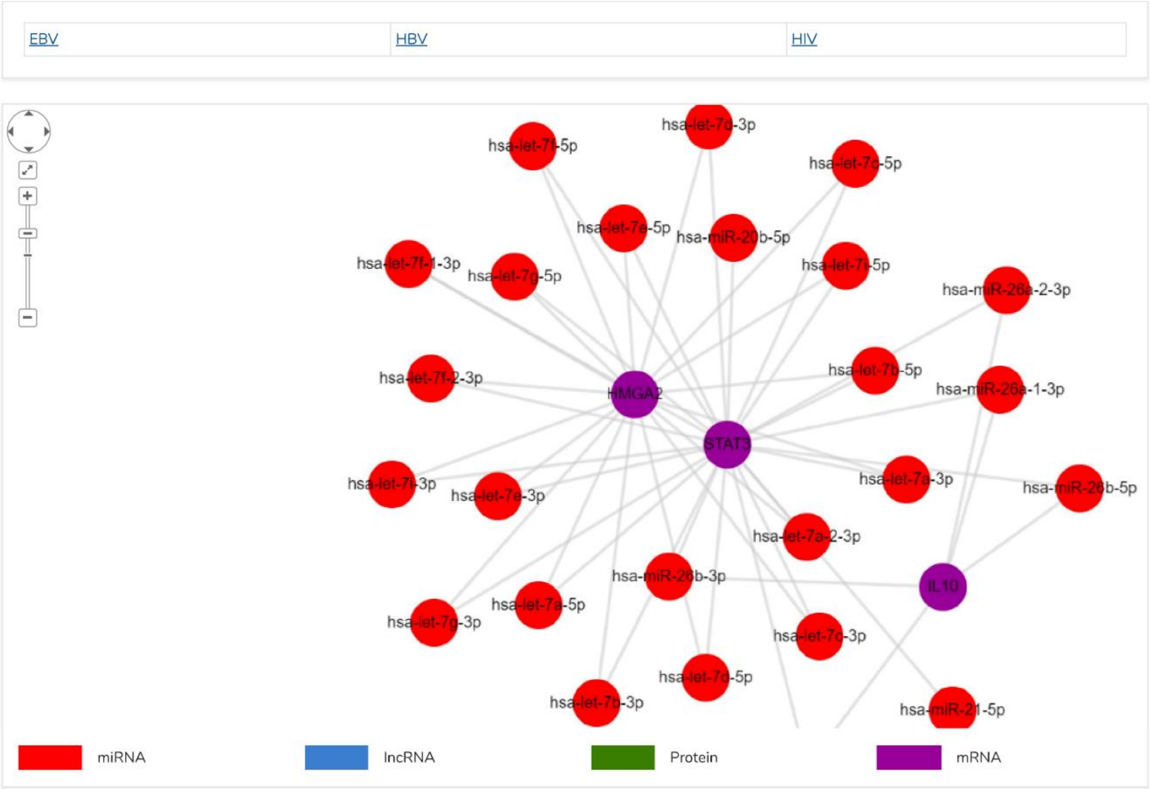

Figure S2. Visualization of virus-host interactions involved in miRNAs.

Figure S3 shows the chromosomal distribution of VISs. We first calculated the chromosome distribution of VIS using the original data. Then we calculated the density score of each chromosome using the following formula:

$$Score_i = \frac{\text{Number of } \ VIS \text{ in } Chromosome_i}{Length_i} \times \sum Length_i$$

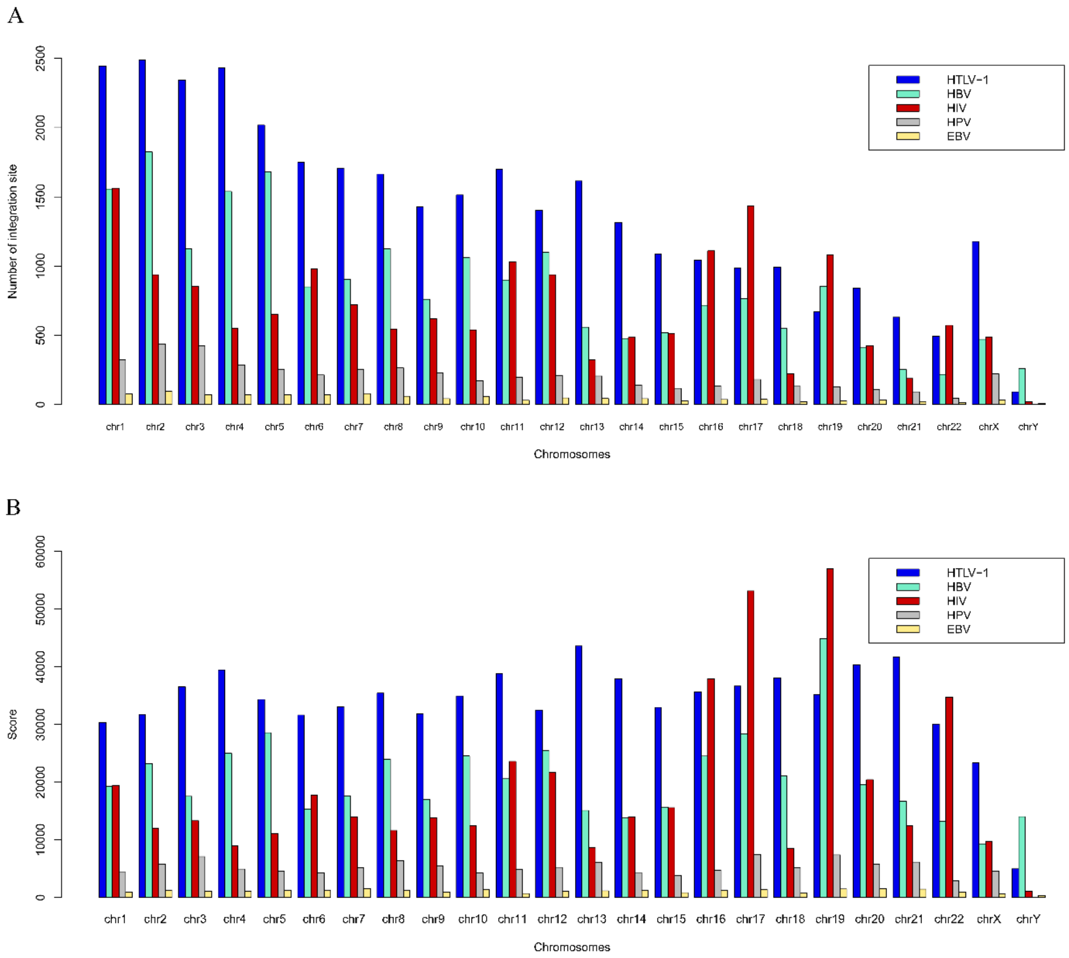

Figure S3. Chromosomal distribution of VISs. (**A**) The original chromosomal distribution of VISs. (**B**) The normalized chromosomal distribution of VISs. The density scores of each chromosome is calculated by dividing VIS number pertained to the chromosome by the ratio of chromosome length to genome length.

Figure S4 is the heatmap of VIS distribution in CFS, we normalize VIS number of 123 fragile sites for HBV, HPV, HIV and EBV with the following formula:

$$N\_VIS_i^v = VIS_i^v * \frac{33842}{N_v}$$

Where $v \in$ {HBV,HPV,HIV,EBV}, VIS_i^v is CFS_i-targeted VIS number of virus v , N_v is the total VIS with precise location.

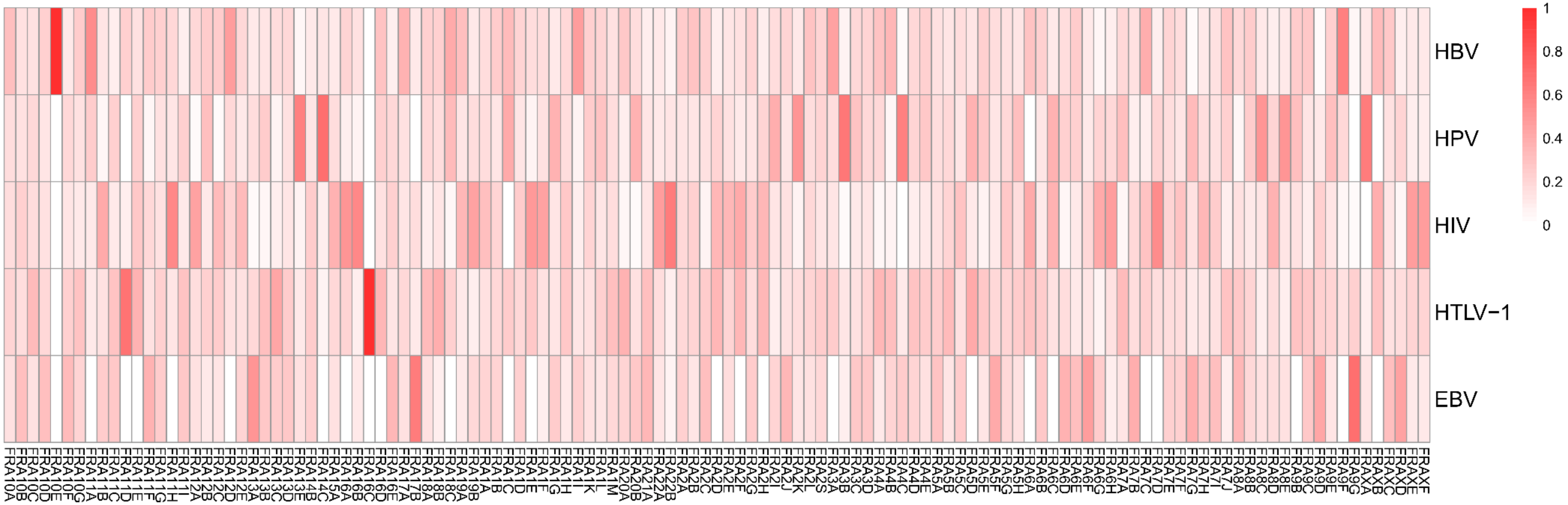

Figure S4. Heatmap of CFSs after normalization of VIS number. VIS numbers of HBV, HPV, HIV and EBV located in each fragile site are normalized by multiplying 33 842 divided by the number of VIS pertained to the virus, where 33 842 is the VIS number of HTLV-1.

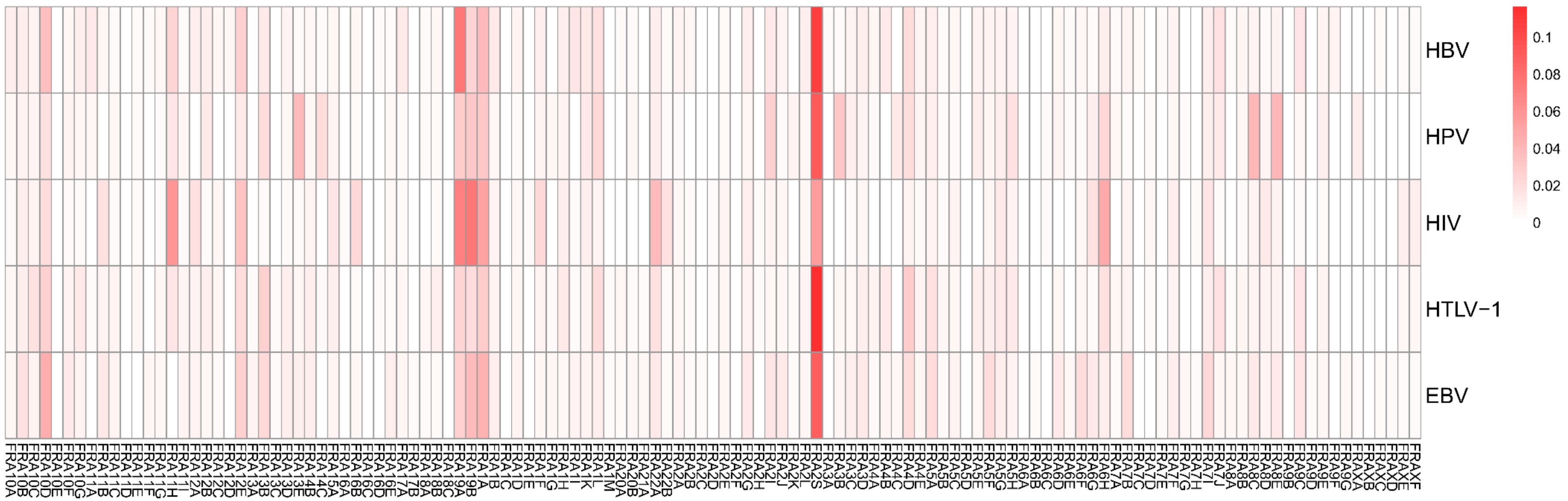

Figure S5. Heatmap of CFS percentage for HBV, HPV, HIV, HITLV-1 and EBV.

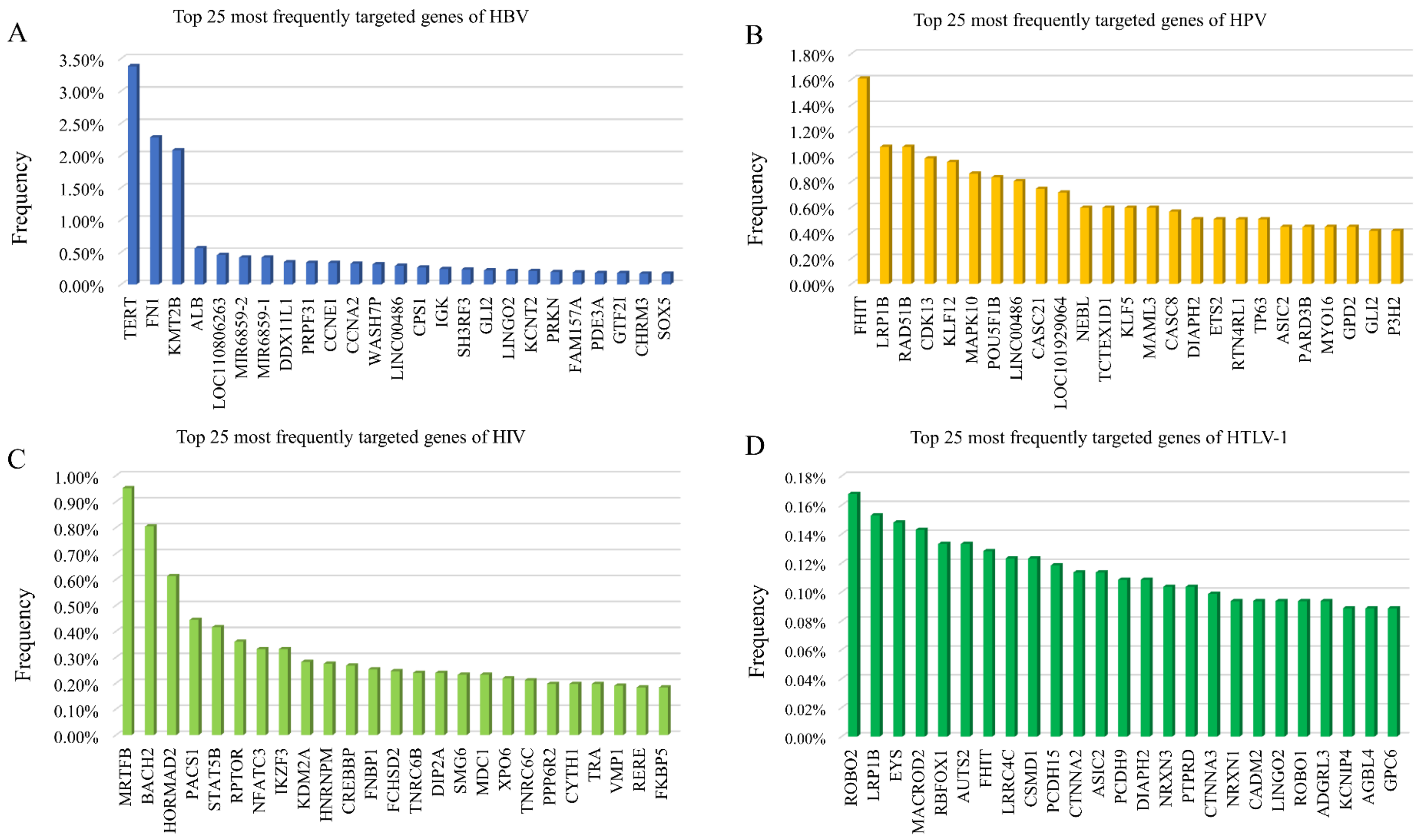

Figure S6. Top 25 most frequently targeted genes of four viruses: HBV (**A**), HPV (**B**), HIV (**C**) and HTLV-1 (**D**).

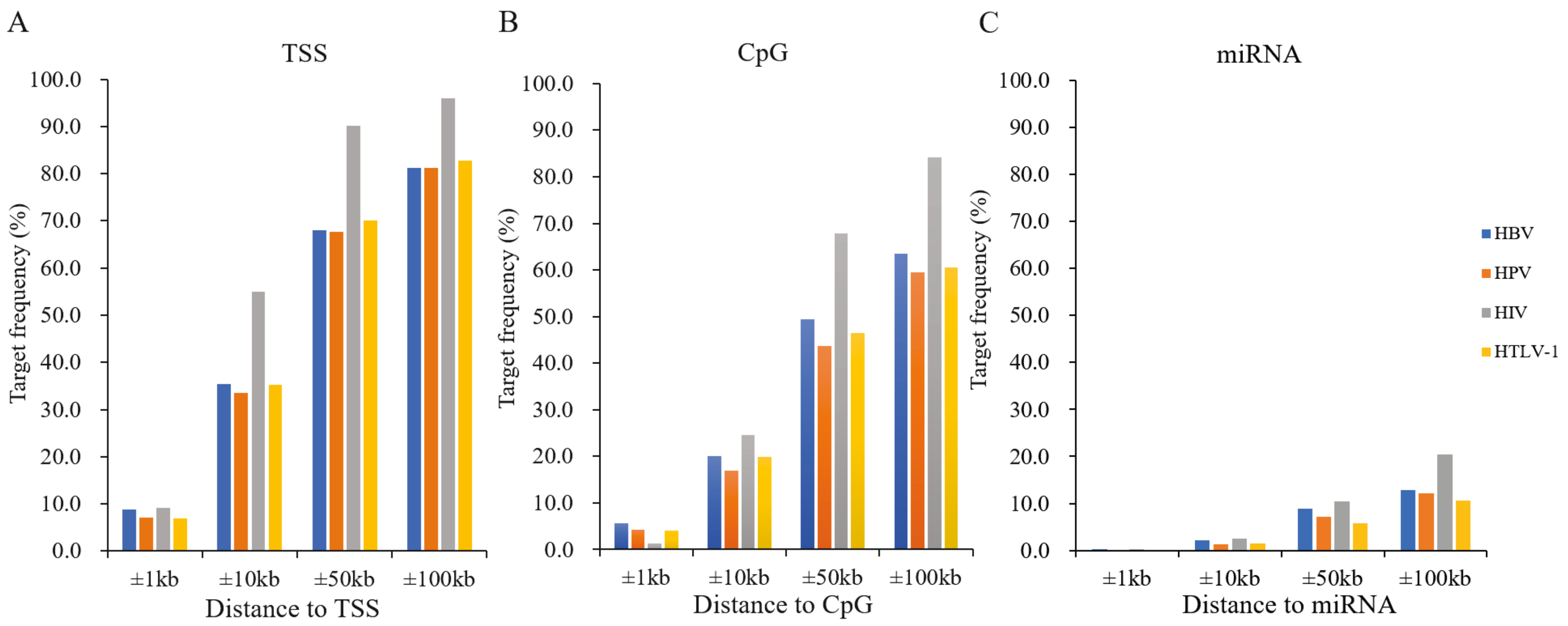

Figure S7. Cellular function regions in the vicinity of VISs. Percentage of VISs from HBV, HPV, HIV and HTLV-1 with respect to different distance are plotted. Both upstream and downstream of VIS near the (**A**) transcription start site (TSS), (**B**) CpG and (**C**) miRNA are calculated.
